# Caterpillar Chewing Vibrations Cause Changes in Plant Hormones and Volatile Emissions in *Arabidopsis thaliana*

**DOI:** 10.3389/fpls.2019.00810

**Published:** 2019-06-26

**Authors:** Mélanie J. A. Body, William C. Neer, Caitlin Vore, Chung-Ho Lin, Danh C. Vu, Jack C. Schultz, Reginald B. Cocroft, Heidi M. Appel

**Affiliations:** ^1^Division of Plant Sciences, Christopher S. Bond Life Sciences Center, University of Missouri, Columbia, MO, United States; ^2^Department of Environmental Sciences, The University of Toledo, Toledo, OH, United States; ^3^Cornell Cooperative Extension Erie County, East Aurora, NY, United States; ^4^School of Natural Resources, University of Missouri, Columbia, MO, United States; ^5^Division of Biological Sciences, University of Missouri, Columbia, MO, United States

**Keywords:** plant defense, herbivory, feeding vibrations, volatile organic compounds, phytohormones

## Abstract

Plant perception of insect feeding involves integration of the multiple signals involved: wounding, oral secretions, and substrate borne feeding vibrations. Although plant responses to wounding and oral secretions have been studied, little is known about how signals from the rapidly transmitted vibrations caused by chewing insect feeding are integrated to produce effects on plant defenses. In this study, we examined whether 24 h of insect feeding vibrations caused changes in levels of phytohormones and volatile organic compounds (VOCs) produced by leaves of *Arabidopsis thaliana* when they were subjected to just feeding vibrations or feeding vibrations and wounding + methyl jasmonate (MeJA), compared to their respective controls of silent sham or wounding + MeJA. We showed that feeding vibrations alone caused a decrease in the concentrations of most phytohormones, compared to those found in control plants receiving no vibrations. When feeding vibrations were combined with wounding and application of MeJA, the results were more complex. For hormones whose levels were induced by wounding and MeJA (jasmonic acid, indole-3-butyric acid), the addition of feeding vibrations caused an even larger response. If the level of hormone was unchanged by wounding and MeJA compared with controls, then the addition of feeding vibrations had little effect. The levels of some VOCs were influenced by the treatments. Feeding vibrations alone caused an increase in β-ionone and decrease in methyl salicylate, and wounding + MeJA alone caused a decrease in benzaldehyde and methyl salicylate. When feeding vibrations were combined with wounding + MeJA, the effects on β-ionone and methyl salicylate were similar to those seen with feeding vibrations alone, and levels of benzaldehyde remained low as seen with wounding + MeJA alone. The widespread downregulation of plant hormones observed in this study is also seen in plant responses to cold, suggesting that membrane fluidity changes and/or downstream signaling may be common to both phenomena.

## Introduction

Like all living organisms, plants must track and respond appropriately to changes in their environment. Plants possess most of the senses found in animals, although usually without the help of specialized organs. For example, light is sensed by individual photoreceptors (Möglich et al., 2010), gravity through subcellular organelles (Massa and Gilroy, 2003), volatiles and other chemicals by an unknown mechanism (Dicke and Baldwin, 2010; Heil, 2014; Turlings and Erb, 2018), and pressure and touch through mechanoreceptors (Braam, 2005; Chehab et al., 2009). We recently demonstrated that plants can also sense and discriminate among vibrations in their environment. Indeed, foliar chemical defenses against insect herbivores are primed following exposure of the leaves to substrate-borne vibrations caused by caterpillar chewing (Appel and Cocroft, 2014).

Plant perception of insect feeding involves integration of the multiple signals involved: wounding, oral secretions, and substrate borne feeding vibrations. Although plant responses to wounding and oral secretions have been studied, little is known about how signals from the rapidly transmitted vibrations caused by chewing insect feeding are integrated to produce effects on plant defenses. The substrate-borne vibrations caused by caterpillar chewing are complex, exhibiting rhythmic patterns in frequency and amplitude (Appel and Cocroft, 2014). These vibrations differ markedly from most previous studies of plant responses to “sound” that use airborne tones as stimuli (e.g., Ghosh et al., 2016, 2017).

Plant chemical defenses are normally present at baseline or constitutive levels that can be induced to higher levels by a current or imminent threat (Karban and Baldwin, 1997). The phytohormone signaling pathways underlying such chemical defense responses to herbivory are relatively well studied and involve interaction among the jasmonate (JA), salicylate (SA), and ethylene (ET) pathways (Pieterse et al., 2009; Smith et al., 2009; Erb et al., 2012). Although they have received less attention in this context, stress hormones (abscisic acid, ABA) and growth regulators (gibberellins, GAs; cytokinins, CKs; auxins, AUXs; brassinosteroids, BRs; etc.) have recently been reported as important players in mediating plant response specificity to herbivory, either directly or *via* the modulation of JA and/or SA pathways (Erb et al., 2012 and references herein). In addition, single, airborne tones can change the expression of genes associated with hormone signaling (Ghosh et al., 2016, 2017). We propose that insect feeding vibrations are likely to elicit changes in the levels of these phytohormones.

While some inducible chemical defenses remain within leaves, some are released into the air as volatile organic compounds (VOCs). These VOCs mediate a diverse array of interactions between plants and insects, including attraction of an herbivore’s natural enemies such as parasitoids and predators (Turlings and Erb, 2018). Changes in VOCs can also prime the defense responses within damaged plants, and between damaged and nearby undamaged plants so that the undamaged tissue reacts more strongly and/or more quickly when subsequently attacked (Frost et al., 2007; Heil and Silva Bueno, 2007a,b). The number of volatile substances in the air around plants can reach several hundreds; however, the blend is often dominated by one or a few major compounds (Schoonhoven et al., 2005). VOC emission from plants under attack can be nearly 2.5-fold higher than that from intact plants and vary in composition with the attacker (Turlings et al., 1990; Takabayashi et al., 1991).

In this study, we asked two questions using the model system of *Arabidopsis thaliana* and substrate borne feeding vibrations caused by *Pieris rapae* caterpillars: (1) do caterpillar feeding vibrations change the phytohormone levels within leaves? and (2) do they influence plant volatile release?

To identify signaling pathways activated by insect feeding vibrations alone, we mechanically delivered feeding vibrations to the plant in the absence of the actual caterpillar so that the other signals arising from insect feeding were absent (Appel and Cocroft, 2014). We measured the levels of foliar 15 phytohormones and 21 VOCs to determine which signaling pathways were up- or down-regulated by feeding vibrations.

## Materials and Methods

### Plant Growth

*Arabidopsis thaliana* (L.) Heynh. (Brassicaceae) Col-0 (wild type) seeds were planted in individual pots (55 × 57 mm), in Pro-Mix potting soil (Premier Horticulture Inc., Quakertown, PA, United States) that was supplemented with 1.8 kg.m^−3^ Osmocote^TM^ slow-release fertilizer (Scotts Company, Marysville, OH, United States). The plants were grown in growth chambers (Percival Scientific Inc., Perry, IA, United States) under the following conditions: temperature of 22°C, 62% relative humidity, 8:16 (L:D) photoperiod, metal halide lamps with a light intensity of 21.9 Watts.m^−2^ (1000Bulbs, Garland, TX, United States).

### Plant Treatments

**FIGURE 1:**
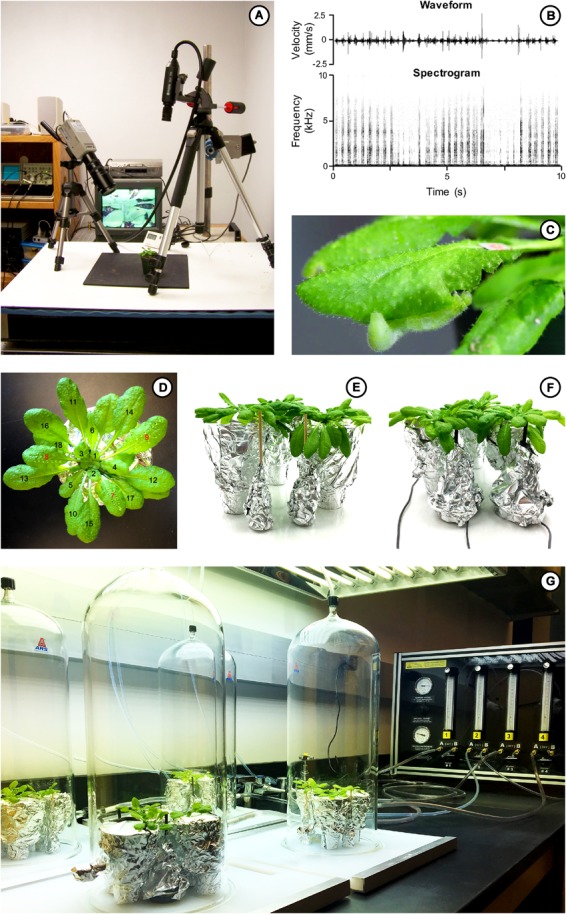
**(A)** Recording *P. rapae* feeding vibrations on *Arabidopsis thaliana* using a laser vibrometer. **(B)** Feeding vibrations of *P. rapae* on *A. thaliana*, (top) waveform, (bottom) spectrogram. **(C)** Fourth-instar *P. rapae* caterpillar feeding on an *A. thaliana* leaf with a reflecting tape for the laser vibrometer to record the chewing vibrations produced. **(D)** Healthy 5–8 week-old pre-reproductive *A. thaliana* with its pot wrapped in aluminum foil to limit VOC emission from exposed soil. Rosette leaves are numbered in ascending order corresponding to age (from young to old), using the youngest leaf larger than 6 mm as the first leaf. Here, leaves 7–9 (red numbers) received mechanical damage by running a pinwheel down both sides of the midrib, and 10 μl of methyl jasmonate (MeJA; 115 mM) onto the wounds. **(E)** A silent sham (no vibration) made from a balsa wood rod is attached to the leaf 7 (target leaf) of each plant. **(F)** Piezoelectric vibrator is attached to the underside of the leaf 7 (target leaf) of each plant to play back to the plant *P. rapae* vibrations previously recorded by Appel and Cocroft (2014). **(G)** Volatile organic compound collection system with three plants for each sample receiving either a silent sham or insect feeding vibration playback for 24 h. **(E)** Laser vibrometer recording vibrations produced by *P. rapae* feeding on *A. thaliana* leaves. **(F)** Vibrations produced by a feeding *P. rapae* caterpillar on *A. thaliana* (Appel and Cocroft, 2014).

Experiments were conducted on healthy, 5–8 week-old pre-reproductive *A. thaliana* (Figure 1). Three plants planted in individual pots were used in each volatile collection chamber to maximize detection of the volatiles released by treatments. Prior to experiments, each pot was wrapped in aluminum foil, covering the exposed soil and the pot to minimize collection of VOCs from these surfaces (Figure 1D–G). The day of the treatment, rosette leaves were numbered using the youngest leaf larger than 6 mm as the first leaf. Leaves were numbered in ascending order corresponding to age (from young to old; Figure 1D) and leaf 7 was designated as the target leaf on each plant to receive one of the vibration treatments. We chose leaf 7 because it was a fully expanded leaf that is large enough to extend over the pot edges and reach the vibration attachment. Plants were either damaged or not prior to the vibration treatment and collection of volatiles. We applied four treatments: (1) no “damage + MeJA” and no vibration, (2) “damage + MeJA” and no vibration, (3) no “damage + MeJA” and vibration, and (4) “damage + MeJA” and vibration. MeJA and other jasmonate derivatives are referred to as “wounding hormones” and are used to elicit chemical defense responses (Yang et al., 2015; Zhang et al., 2015; Fedderwitz et al., 2016; Lundborg et al., 2016; Tianzi et al., 2018). Treatments were as follow.

#### Damage + MeJA

The target leaf and two other leaves (leaves 7, 8, 9) were mechanically damaged prior to volatile collection (Figure 1D; *N* = 12 sets of three plants for phytohormones and 22 for VOCs). Approximately 30 min before starting VOC collection (Figure 1G), a pinwheel was run down both sides of the midrib creating two lines of damage on each leaf and 10 μl of methyl jasmonate (MeJA; 115 mM) were applied to each line of damage directly after damage (Figure 1D).

#### Feeding Vibrations

Feeding vibrations of *P. rapae* caterpillar (Lepidoptera, Pieridae) recorded by Appel and Cocroft (2014) (Figure 1A–C) were played back to plants for 24 h (*N* = 12 sets of three plants for phytohormones and 24 for VOCs). Playbacks were conducted using 2-inch audio speakers (8 ohms, 0.5 W), modified as in Michael et al. (2019) to minimize production of airborne sound, and with a dowel attached to the coil to allow coupling of vibrations to a leaf (Figure 1F). The speakers were driven using stereo amplifiers (Dayton Audio, DTA1). The target playback leaf on each plant was attached to the dowel with wax using gentle pressure to avoid damaging the leaf (clear, unscented HoldingWax^TM^, that is similar to accelerometer mounting wax). Each of the three plants in a chamber was contacted by a different, independently driven speaker contacting the leaf with wax (Figure 1G). Speakers were covered with aluminum foil to minimize volatile release from their surfaces (Figure 1F,G). Twelve 10-s exemplars were used for playbacks (Figure 1B), with each exemplar drawn from a laser vibrometer recording of a different *P. rapae* caterpillar on a different *Arabidopsis* plant (Figure 1A–C; see Appel and Cocroft, 2014 for details). The playbacks had a peak amplitude of 2.5 mm/s, and the playback exemplars were pre-filtered as in Cocroft et al. (2014) to compensate for the frequency response of each of the six modified speakers.

#### Damage + MeJA + Feeding Vibrations

Approximately 30 min before starting VOC collection, plants received damage, MeJA, and vibration playback started at the same time as VOC collection, as described above (*N* = 12 sets of three plants for phytohormones and 24 for VOCs).

#### No Damage + No MeJA + Silent Sham

These plants received no “damage + MeJA” treatment, and no vibration playback (*N* = 12 sets of three plants for phytohormones and 24 for VOCs). A silent sham provided a control for any effect of the attachment of the vibration playbacks on plant responses. The silent sham consisted of a dowel resting on a foil-covered rubber base and attached to the leaf as described above for the modified speakers (Figure 1E; no vibrations). One leaf from each of the three plants in a chamber was contacted by its own silent sham (Figure 1G).

### Experimental Design

Plants were first treated with or without damage + MeJA. Then, the VOC collection took place for 24 h (pool of three plants for each sample to reach the limit of detection, each set of three plants constituted one data point for all treatments; Figure 1G) while plants were treated with either insect feeding vibrations or silent shams. After 24 h, plants were removed from the volatile collection chambers and photographed to measure leaf area, and then the leaves were harvested at the base of their petiole for hormone analysis, flash frozen in liquid nitrogen and stored at −80°C. To obtain enough material for hormone analysis, leaves were pooled and one sample consisted of 9 leaves from each of the three plants in the chamber (leaves 4–12 as described above).

### Phytohormone Analysis

**FIGURE 2:**
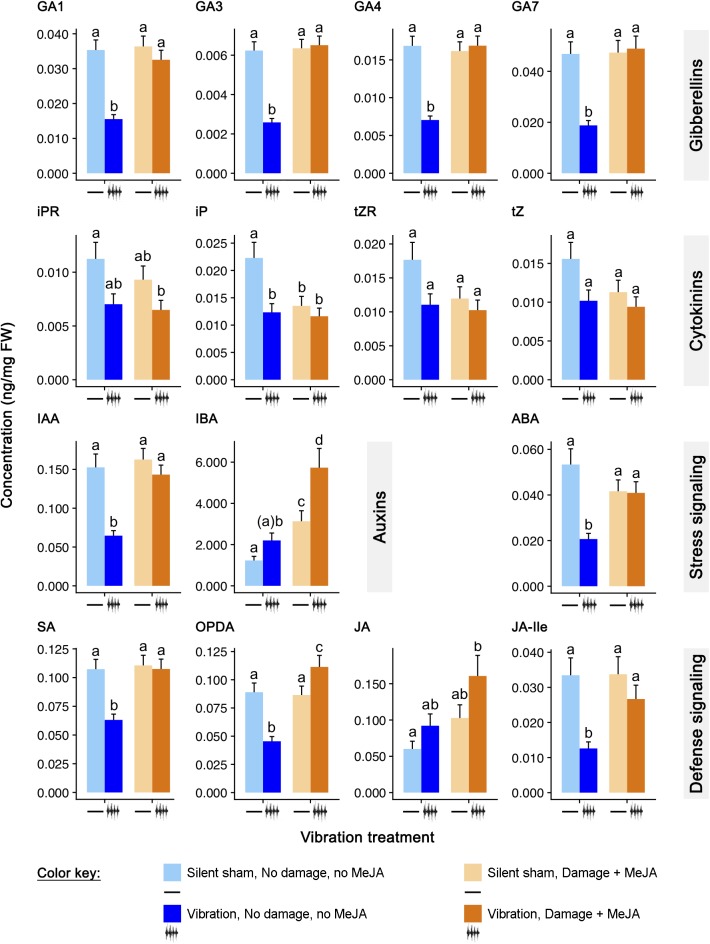
Effects of feeding vibrations on phytohormone signaling pattern. Concentrations (ng/mg FW; least squares mean ± S.E.) of phytohormones differing between treatments. “Round” (the set of plants tested at the same time) was included as a random effect. All *p*-Values have been adjusted for testing of multiple hormones, using the FDR procedure of Benjamini and Hochberg (1995). Statistical differences (*p*-Value ≤ 0.05) between different treatments are shown by different letters (a–c). See Table 1 for statistical details. *N* = 12 sets of three plants for phytohormones.

A panel of 15 phytohormones was measured (Figure 2). Three were jasmonates known to be involved in plant responses to insect herbivores: JA, a jasmonic acid precursor *cis*-(+)-12-oxo-phytodienoic acid (OPDA), and the conjugate jasmonoyl-isoleucine (JA-Ile) (Pieterse et al., 2009; Schuman et al., 2018; Wasternack and Feussner, 2018). Salicylic acid (SA) is a phytohormone involved in plant defense signaling against biotrophic pathogens (Dempsey et al., 2011) and fluid-feeding herbivores (Erb et al., 2012; Pieterse et al., 2012; Thaler et al., 2012). Abscisic acid (ABA) has a critical role in plant responses to environmental stresses, including drought, cold/freezing tolerance, and heat stress (Finkelstein, 2013). We measured two forms of auxin (AUX), a phytohormone that is involved in almost every facet of plant life (Zhao, 2014): indole-3-acetic acid (IAA) and indole-3-butyric acid (IBA). Four forms of gibberellins, a class of phytohormones that modulates growth and development (Sun, 2008) were also measured: gibberellin 1 (GA1), gibberellic acid (GA3), gibberellin 4 (GA4), and gibberellin 7 (GA7). And last, we measured four forms of cytokinins, a class of phytohormones responsible for cell division and growth and involved in many plant responses to stress (Kieber and Schaller, 2014): 6-(Δ2-isopentenyl) adenine riboside (iPR), 6-(Δ2-isopentenyl) adenine (iP), *trans*-zeatin riboside (tZR), and *trans*-zeatin (tZ).

#### Phytohormone Extraction

Phytohormones were analyzed following Body et al. (2019) protocol. Briefly, phytohormones [(*defense signaling*) SA, OPDA, JA, JA-Ile; (*stress signaling*) ABA; (*gibberellins*) GA1, GA3, GA4, GA7; (*cytokinins*) iPR, iP, tZR, tZ; (*auxins*) IAA, IBA] were extracted from 200 mg of frozen leaf powder with 2 ml of extraction solvent, 2-propanol:H_2_O:concentrated HCl (2:1:0.002, vol:vol:vol) and 50 μl of the working solution of internal standards (mix of two stable isotope-labeled compounds, D6-ABA, [2H6]-cis, *trans*-abscisic acid and D6-iP, [2H6]-N6-isopentenyladenine, at 500 ppb) at 80 r.p.m. for 30 min. After adding 1 ml dichloromethane, samples were shaken again for 30 min and centrifuged at 13, 000 *g* for 5 min. After centrifugation, two phases formed; plant debris was between the two layers. The lower phase was transferred into a 2 ml Eppendorf tube and concentrated under a gentle nitrogen gas flow, while a second extraction was performed by adding 1 ml dichloromethane to each sample and repeating the same steps as described above. After concentration under nitrogen gas flow, samples were then dissolved in 100 μl methanol and transferred into a 150 μl insert in a 2 ml LCMS-certified amber glass vials (Waters, Milford, MA, United States).

#### HPLC-ESI-MS/MS Conditions

Thirty μl of sample solution were injected twice – one for positive ion, one for negative ion (Body et al., 2019) – into a high-performance liquid chromatography electron spray ionization tandem mass spectrometer (HPLC-ESI-MS/MS) for quantification of all phytohormones. The phytohormones were separated by a reverse-phase C_18_ HPLC column (Kinetex 2.6 μm C18 100 Å, LC column 100 × 4.6 mm) (Phenomenex, Torrance, CA, United States) on a Waters 2695 HPLC (Milford, MA, United States) system coupled with a UV detector (Waters 996 Photodiode Array Detector) and a Waters Acquity TQ triple quadrupole mass spectrometer (MS/MS) detector (Waters TQ Detector, Acquity Ultra Performance LC), controlled by the Waters Acquity TQ Detector (ACQ-TQD) software (version 1.40.2335). The optimized parameters (mobile phase, solvent flow, oven temperature, precursor and product ions, retention time, collision energy, and capillary voltage) for our instrument are presented in Body et al. (2019).

### Analysis of Volatile Organic Compounds

Most studies of plant VOCs use a semi-quantitative method based on the area of peaks obtained by gas chromatography. Peak area may not be an accurate measure of the amount of an individual compound because individual compounds do not interact with the chromatographic stationary phase in the same way and the detector response of the compound often is not one-to-one linear with respect to the concentration, peak area may not be an accurate measure of the amount of an individual compound. In a preliminary experiment reported in Supplement 1, we compared the results of VOC measurement expressed as peak area to those expressed as actual amounts based on a standard curve of commercially available volatile compounds. VOCs emitted by *A. thaliana* and *Brassica oleracea* with and without feeding by *Pieris rapae* caterpillars were collected and analyzed by gas chromatography - mass spectrometry (GC-MS). We found that the results were qualitatively similar for both methods. When an individual compound was induced by caterpillar feeding that induction was detected by both methods. However, the two methods were not quantitatively similar. The peak area method underestimated the amount of dimethyl sulfide and overestimated the amount of *cis*-3-hexenyl acetate, (S)-(-)-limonene, α-pinene, and methyl salicylate compared to the standard curve method (Supplement 1). As a result, in this study we chose to measure only those compounds that we could quantify with commercially available standards.

#### VOC Collection

A flow-through volatile collection system (Figure 1G; Analytical Research Systems, Inc.) consisting of a non-humidified 4-channel air delivery system (VCS-ADS-4AFM-2C) with 3-Stage Air Filtration System (ADS-3STPR-AFS) and an internal vacuum pump (MVCS-VAC-PUMP) with four glass chambers each with two sampling ports (VCC-G6X12-NL-2P; 15 cm diameter × 30 cm high) was used to collect VOCs. Three plants of the same treatment were placed under the same collection chamber to maximize detection of VOCs (Figure 1). Each chamber implemented a different treatment (sham, damage only, vibration only, or damage + vibration). VOCs were collected for 24 h under the following conditions: temperature of 22°C, 24:0 h (L:D) photoperiod, grow lamps (Sun Blaze^®^T5 fluorescent, 4 ft; Sunlight Supply, Wixom, MI, United States) with a light intensity of 48.6 Watts.m^−2^, using an airflow of 0.25 unit of atmospheric air, with a vacuum pressure of 25 mmHg. A trap (90 mg 20:35 mesh Tenax-TA/Carboxen 1000/Carbosieve SIII) was attached to the outlets. Prior to use, these traps were conditioned at 300°C for 30 min.

#### VOC Standards

All VOC standards were purchased from Sigma-Aldrich (St. Louis, MO, United States): 1-penten-3-ol, 1-penten-3-one, 3-pentanone, α-caryophyllene, α-farnesene, α-pinene, β-caryophyllene, β-farnesene, β-ionone, benzaldehyde, *cis*-3-hexen-1-ol, *cis*-3-hexenyl acetate, eugenol, hexyl acetate, jasmone, limonene, linalool, methyl jasmonate, methyl salicylate, ocimene, *trans*-2-hexen-1-al. All VOC standards were diluted in 100% methanol.

#### GC-MS Analysis

Volatile organic compounds were analyzed by gas chromatrography – mass spectrometry (GC-MS) following a quantitative protocol we developed (Supplement 1). VOCs were thermally desorbed in CDS 7500 Thermal Desorption Autosampler (CDS Analytical Inc., Oxford, PA, United States) at 300°C for 5 min. Helium was used as carrier gas at a flow rate of 20 ml/min. After desorption, the analytes were released to a focusing trap in CDS Dynatherm 9300 ACEM. The temperature of the trap initially set at 45°C was raised to 200°C. All the analytes were then transferred to Agilent 6890N gas chromatograph (Agilent, Santa Clara, CA, United States) through a transfer line set at 225°C. The GC installed with a DB-5MS column (30 m × 0.25 mm I.D.; Agilent J&W, Santa Clara, CA, United States) was interfaced to an Agilent 5973 quadrupole mass spectrometer. The GC column was initially set at 35°C for 10 min, then increased to 200°C at 10°C/min, and to 260°C at 3°C/min. After reaching 260°C, the temperature was held for 6 min. The split injection was used with split ratio of 5:1 and the carrier gas flow of 1.0 ml/min. Injector temperature was set at 275°C, transfer line between the GC and mass spectrometer was held at 150°C. The MS source was held at 230°C. The emission current was 40 μ/amps, with a maximum ionization time of 25,000 μ/s, and the ion scan range 50–400 m/z, the ion storage level 45 m/z and the pre-scan ionization time 100 μ/s. The optimized parameters (retention time and quantification ions) for our instrument are presented in Table 1.

**Table 1:** List of 21 volatile organic compounds (VOCs) analyzed in the headspace of *Arabidopsis thaliana* rosettes.

No.	VOCs	RT	Quantification ions	LOD	Slope	R^2^
1	1-penten-3-ol	3.28	57^∗^, 29, 27, 31, 41	0.0125	*y* = 66.97x + 338.4	0.376
2	1-penten-3-one	3.30	55^∗^, 27, 84, 29, 57	0.0016	*y* = 17.33x + 587	0.452
3	3-pentanone	3.41	57^∗^, 29, 86, 27, 28	0.0096	NA	NA
4	*trans*-2-hexen-1-al	5.55	41, 42, 39, 83, 69^∗^	0.0229	*y* = 2445x – 15070	0.907
5	*cis*-3-hexen-1-ol	5.60	67^∗^, 41, 39, 55, 82	0.0123	*y* = 8145x – 26040	0.968
6	α-pinene	6.77	93^∗^, 92, 91, 77, 79	0.0192	*y* = 16170x – 91960	0.788
7	benzaldehyde	7.12	77^∗^, 106, 105, 51, 50	0.0038	*y* = 10770x – 21410	0.981
8	*cis*-3-hexenyl acetate	7.61	43, 67^∗^, 82, 41, 39	0.0029	*y* = 15960x – 44720	0.985
9	hexyl acetate	7.63	43, 56^∗^, 55, 61, 42	0.0037	*y* = 7434x – 15740	0.985
10	limonene	7.95	68, 93^∗^, 39, 67, 41	0.0025	*y* = 11440x – 10720	0.988
11	ocimene	8.12	93^∗^, 91, 79, 80, 77	0.0035	*y* = 11920x – 18960	0.986
12	linalool	8.76	71^∗^, 93, 55, 43, 41	0.0165	*y* = 6072x – 51970	0.861
13	methyl salicylate	9.83	120^∗^, 92, 152, 121, 65	0.0132	*y* = 22500x – 105500	0.976
14	eugenol	11.37	164^∗^, 103, 77, 149, 131	0.0085	*y* = 20820x – 125200	0.938
15	jasmone	11.77	164^∗^, 79, 110, 149, 122	0.0205	*y* = 6967x – 25280	0.957
16	β-caryophyllene	12.06	93^∗^, 133, 91, 41, 79	0.0046	*y* = 8006x – 28150	0.977
17	β-farnesene	12.21	41, 69^∗^, 93, 67, 79	0.0183	*y* = 878.9x – 4720	0.944
18	α-caryophyllene	12.37	93^∗^, 80, 41, 121, 92	0.0004	*y* = 8006x – 28150	0.977
19	β-ionone	12.51	177^∗^, 43, 91, 135, 178	0.0013	*y* = 36610x – 138700	0.944
20	α-farnesene	12.73	41, 93^∗^, 69, 55, 107	0.0256	*y* = 1121x – 2638	0.954
21	methyl jasmonate	13.83	83^∗^, 41, 151, 67, 95	0.0231	*y* = 6520x – 40320	0.889

*RT, retention time (min); LOD, limit of detection (μg/m^3^); R^2^, coefficient of determination. For the slope, y, response, x, concentration. ^∗^Indicates quantitative ions.*

All plant volatile data were individually checked for peak identification. Valid peaks for a particular compound had to be at least three times above background and contain the appropriate diagnostic ion fragments. Validated peaks were then expressed as ppm per plant area per hour, using plant area obtained as described below.

#### Leaf Area Analysis

Since volatile emissions are likely to depend on the surface area of plant tissue, leaf area was determined from photos of the plants taken at the end of the VOC collection using ImageJ version 1.49 m software (National Institutes of Health, United States) and the Fiji plugin. A ruler placed by the plant was used to set the scale and leaf surface was calculated by outlining the surface of interest. VOC concentrations were then expressed as an amount per leaf area per time unit.

### Statistical Analysis

Phytohormones and VOCs were analyzed using a general linear mixed (GLM) model, with a gamma distribution; models included “vibration,” “damage + MeJA,” and “vibration × damage + MeJA” as fixed effects, and “round” (the set of plants treated at the same time) as a random effect. For the VOCs, the 24 rounds were conducted in two groups of 12 with an interval of 2 months. For analysis, possible differences between the two sets of 12 rounds were accounted for by including a third term (“experiment,” indicating one set of 12 rounds) as a fixed effect to allow testing for possible interactions with the other variables (i.e., whether the effect of the vibration and damage treatments differed between the two sets of 12 rounds). Since the results of the two experiments did not differ for β-ionone, hexyl acetate, and benzaldehyde, data from both experiments were combined, and we reported statistics for the combined data. MeSA emission was above the limit of detection only in one of the experiments.

Statistical analyses were conducted in SAS v. 9.4. For the VOCs, only the compounds detected in 50% or more of the rounds were analyzed. The resulting *p*-Values were adjusted using the FDR procedure of Benjamini and Hochberg (1995) for the use of multiple response variables (fifteen phytohormones, four VOCs); *p*-Values were similarly adjusted in post-hoc analyses to account for multiple comparisons.

## Results

### Phytohormones

Levels of OPDA, JA, and JA-Ile were significantly influenced by “damage + MeJA” alone, vibration alone, and the interaction of the two treatments (Figure 2 and Table 2). Vibration in the absence of “damage + MeJA” reduced the levels of OPDA and JA-Ile to 51% and 38% of the levels of control plants, respectively, whereas levels of JA were unchanged. Consistent with our previous work showing vibration priming of chemical defenses, vibration in addition to “damage + MeJA” caused higher levels of OPDA and JA than did “damage + MeJA” in the absence of vibration. The level of JA-Ile in “damage + MeJA” treated plants was unaffected by feeding vibrations.

**Table 2:** The effect of vibration and damage + MeJA on phytohormone levels, based on a general linear mixed model.

Variable	Treatment	d.*f*.	*F* Value	Pr > *F*
***Gibberellins***				
	Vibration	1, 32	75.42	0.000^∗∗∗^
GA1	Damage + MeJA	1, 32	51.16	0.000^∗∗∗^
	Vibration ∗ Damage + MeJA	1, 32	43.81	0.001^∗∗∗^
	Vibration	1, 31	48.06	0.000^∗∗∗^
GA3	Damage + MeJA	1, 31	58.13	0.000^∗∗∗^
	Vibration ∗ Damage + MeJA	1, 31	53.77	0.000^∗∗∗^
	Vibration	1, 33	44.95	0.000^∗∗∗^
GA4	Damage + MeJA	1, 33	45.12	0.000^∗∗∗^
	Vibration ∗ Damage + MeJA	1, 33	54.67	0.000^∗∗∗^
	Vibration	1, 33	23.35	0.000^∗∗∗^
GA7	Damage + MeJA	1, 33	28.17	0.000^∗∗∗^
	Vibration ∗ Damage + MeJA	1, 33	26.90	0.000^∗∗∗^
***Cytokinins***				
	Vibration	1, 44	8.39	0.007^∗∗^
iP	Damage + MeJA	1, 44	4.76	0.047^∗^
	Vibration ∗ Damage + MeJA	1, 44	2.95	0.140
	Vibration	1, 33	9.48	0.006^∗∗^
iPR	Damage + MeJA	1, 33	0.98	0.329
	Vibration ∗ Damage + MeJA	1, 33	0.17	0.788
	Vibration	1, 33	5.16	0.032^∗^
tZ	Damage + MeJA	1, 33	2.24	0.154
	Vibration ∗ Damage + MeJA	1, 33	0.82	0.464
	Vibration	1, 33	4.79	0.036^∗^
tZR	Damage + MeJA	1, 33	2.67	0.129
	Vibration ∗ Damage + MeJA	1, 33	1.22	0.378
***Auxins***				
	Vibration	1, 22	38.46	0.000^∗∗∗^
IAA	Damage + MeJA	1, 22	27.36	0.000^∗∗∗^
	Vibration ∗ Damage + MeJA	1, 22	21.02	0.000^∗∗∗^
	Vibration	1, 44	13.30	0.001^∗∗∗^
IBA	Damage + MeJA	1, 44	33.78	0.000^∗∗∗^
	Vibration ∗ Damage + MeJA	1, 44	0.00	0.952
***Stress signaling***				
	Vibration	1, 31	21.98	0.000 ^∗∗∗^
ABA	Damage + MeJA	1, 31	4.45	0.054^∙^
	Vibration ∗ Damage + MeJA	1, 31	20.45	0.000 ^∗∗∗^
***Defense signaling***				
	Vibration	1, 33	18.84	0.000^∗∗∗^
SA	Damage + MeJA	1, 33	19.03	0.000^∗∗∗^
	Vibration ∗ Damage + MeJA	1, 33	15.16	0.001^∗∗∗^
	Vibration	1, 33	9.31	0.006^∗∗^
OPDA	Damage + MeJA	1, 33	40.18	0.000^∗∗∗^
	Vibration ∗ Damage + MeJA	1, 33	45.65	0.000^∗∗∗^
	Vibration	1, 33	6.49	0.018^∗^
JA	Damage + MeJA	1, 33	10.16	0.005^∗∗^
	Vibration ∗ Damage + MeJA	1, 33	0.00	0.952
	Vibration	1, 33	16.80	0.001^∗∗∗^
JA-Ile	Damage + MeJA	1, 33	6.60	0.022^∗^
	Vibration ∗ Damage + MeJA	1, 33	6.29	0.029^∗^

*“Round” (the set of plants tested at the same time) was included as a random effect. All p-Values have been adjusted for testing of multiple hormones, using the FDR procedure of Benjamini and Hochberg (1995). Significant effects are shown as follow: ^∗∗∗^when p-Value ≤ 0.001; ^∗∗^when p-Value ≤ 0.01; ^∗^when p-Value ≤ 0.05; ^∙^when p-Value ≤ 0.10. N = 12 sets of three plants for phytohormones. d.f., degree of freedom; Pr > F, Probability of the F statistic. See section “Materials and Methods” for full name of phytohormones.*

Levels of SA were significantly influenced by “damage + MeJA” alone, vibration alone, and the interaction of the two treatments (Figure 2 and Table 2). Vibration alone caused levels of SA to decrease to 59% of levels in control plants.

Levels of ABA were also significantly influenced by “damage + MeJA” alone, vibration alone, and the interaction of the two treatments (Figure 2 and Table 2). Vibration alone caused a decrease in the levels of ABA to 39% of levels in control plants.

Levels of IAA were significantly influenced by “damage + MeJA” alone, vibration alone, and the interaction of the two treatments (Figure 2 and Table 2). Vibration alone caused a decrease in the levels of IAA to 42% of levels in control plants. Levels of IBA were significantly increased by “damage + MeJA” alone and vibration alone but there was no significant interaction of the two treatments (Figure 2 and Table 2). Vibration alone had no effect on the levels of IBA.

Levels of all GAs (GA1, GA3, GA4, and GA7) were significantly influenced by “damage + MeJA” alone, vibration alone, and the interaction of the two treatments (Figure 2 and Table 2). Vibration alone caused decreases in the levels of GA1 (44%), GA3 (42%), GA4 (42%), and GA7 (40%) compared to the levels in control plants.

Levels of all CKs (iPR, iP, tZR, and tZ) were significantly influenced by vibration alone, and for iP there was also an interaction with “damage + MeJA” (Figure 2 and Table 2). Vibration alone caused decreases in the concentrations of all four cytokinins, although those decreases were statistically significant only for iP which was present at only 55% of the levels in control plants.

### Volatile Organic Compounds

Of the 21 compounds investigated (Table 1), there were only four compounds only that met the detection criteria of a signal three times above background noise and ion fragmentation that matched commercial standards for at least half of the samples in one or both of the replicate experiments. These were β-ionone, hexyl acetate, benzaldehyde, and methyl salicylate (MeSA), and results for the 18 other VOCs are therefore not presented here.

**FIGURE 3:**
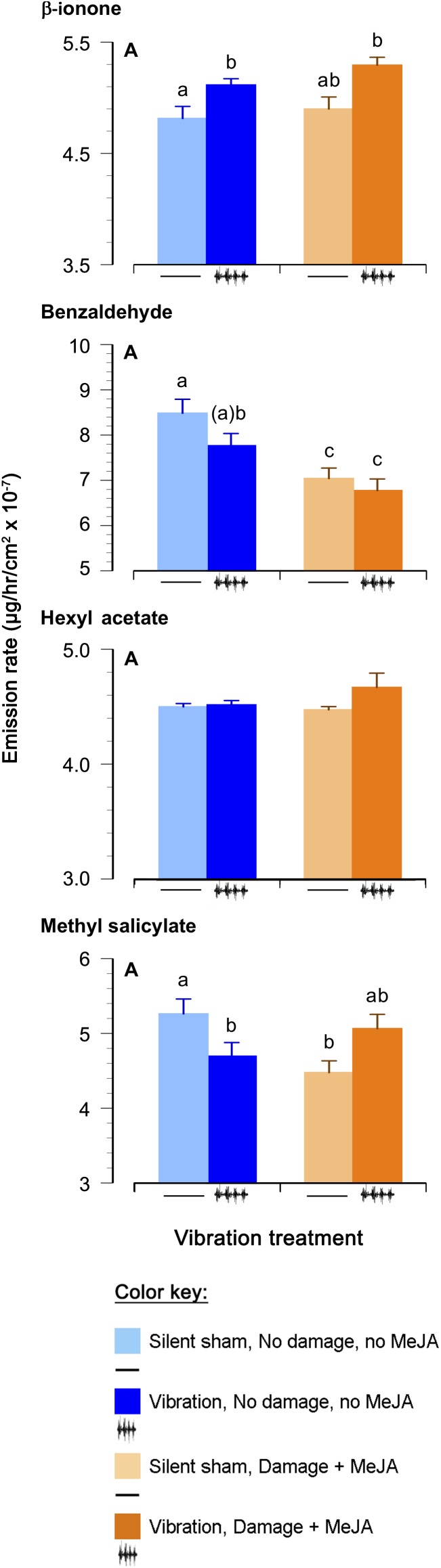
Effects of feeding vibrations on release of plant volatiles. Emission rate (μg/hr/cm^2^ × 10^−7^; least squares mean ± S.E.) of volatile organic compounds differing between treatments. “Round” (the set of plants tested at the same time) was included as a random effect, and *p*-Values have been adjusted for testing of multiple compounds, as above. Statistical differences (*p*-Value ≤ 0.05) between different treatments are shown by different letters (a–c). See Table 2 for statistical details. *N* = 22 for the Damage + MeJA treatment and 24 for all the other treatments.

Only levels of β-ionone, benzaldehyde, and methyl salicylate were significantly influenced by one or more of the treatments (Figure 3 and Table 3). Vibration increased the emission of β-ionone when the plants received just vibrations or vibrations and “damage + MeJA” (5 and 7%, respectively) (Figure 3 and Table 3).

**Table 3:** The effect of vibration and damage on volatile concentrations, based on a general linear mixed model with a gamma distribution.

Variable	Treatment	d.*f*.	*F*-value	Pr > *F*
	Vibration	1, 86	14.96	0.000^∗∗∗^
β-ionone	Damage + MeJA	1, 86	2.12	0.199
	Vibration ∗ Damage + MeJA	1, 86	0.22	0.638
	Vibration	1, 64	5.58	0.042ˆ*
Benzaldehyde	Damage + MeJA	1, 64	24.40	0.000^∗∗∗^
	Vibration ∗ Damage + MeJA	1, 64	0.53	0.624
	Vibration	1, 64	2.38	0.171
Hexyl acetate	Damage + MeJA	1, 64	1.10	0.299
	Vibration ∗ Damage + MeJA	1, 64	2.32	0.266
	Vibration	1, 33	0.02	0.889
Methyl salicylate	Damage + MeJA	1, 33	3.75	0.123
	Vibration ∗ Damage + MeJA	1, 33	6.36	0.067^∙^

*“Round” (the set of plants tested at the same time) was included as a random effect. All p-Values have been adjusted for testing of multiple compounds, using the FDR procedure of Benjamini and Hochberg (1995). Statistical effects are shown as follow: ^∗∗∗^when p-Value ≤ 0.001; ^∗^when p-Value ≤ 0.05; ^∙^when p-Value ≤ 0.10. Data are represented as average ± S.E. N = 22 for the Damage + MeJA treatment and 24 for all the other treatments. d.f., degree of freedom; Pr > F, Probability of the F statistic.*

In contrast, vibration decreased the levels of benzaldehyde in undamaged plants (8%), and “damage + MeJA” caused an even larger decrease (18%), independent of vibration (Figure 3 and Table 3). There was no interaction between “damage + MeJA” and vibration treatments in levels of benzaldehyde (Figure 3 and Table 3).

Vibration decreased the levels of MeSA in undamaged plants (11%) and levels were similarly lower for “damage + MeJA” plants (Figure 3 and Table 3). However, plants that received vibration in addition to “damage + MeJA” had values similar to those of controls receiving no vibration and no “damage + MeJA” (Figure 3 and Table 3). In other words, the effect of vibration on MeSA depended on whether or not plants also received damage + MeJA.

## Discussion

In this study, we investigated the impact of substrate transmitted recordings of feeding vibrations caused by *P. rapae* caterpillars on phytohormone and VOC profiles of *A. thaliana* plants. We observed that feeding vibrations alone led to a significant increase of JA, IBA, and β-ionone concentrations, and a significant decrease of OPDA, JA-Ile, SA, ABA, IAA, GA1, GA3, GA4, GA7, iP, iPR, benzaldehyde, and MeSA concentrations. When plants were pre-treated with “damage + MeJA,” feeding vibrations led to a significant increase of OPDA, JA, IBA, β-ionone, and MeSA concentrations, and a significant decrease of iPR concentration.

### Extensive Changes in Phytohormone Signaling

Plant responses to insects and wounding require the oxylipin/JA pathway (Pieterse et al., 2009; Wasternack and Feussner, 2018). The direction of change in response to feeding vibrations differed among OPDA, JA, and JA-Ile in this study, which could be due to their dynamic and time dependent metabolism, as has been reported elsewhere (Wasternack and Feussner, 2018). Fluctuating levels of JA were reported by Ghosh et al. (2016) for 6, 24, and 48 h after *A. thaliana* plants experienced a single, airborne tone for 1 h. They reported no difference in levels of JA at 6 h, a decrease in JA at 24 h, and an increase in JA at 48 h. Ethylene (ET) was not measured in this study, but could be one source of variation in JA responses to vibration. Indeed, ET is known to be released by leaf damage and to modulate signaling in the JA pathway (Pieterse et al., 2009; Rehrig et al., 2014). When “damage + MeJA” was added to our vibration treatment, the level of JA was much higher than with vibration alone, suggesting that vibrations potentiated the JA response, as previously reported for glucosinolates and anthocyanins (Appel and Cocroft, 2014). Alternatively, MeJA may have been converted back to JA to interact with other metabolic pathways involved in defense responses (Koo et al., 2013; Jang et al., 2014).

The observed decrease in levels of both SA and MeSA in response to caterpillar feeding vibrations does not support a model in which they are readily interconverted *in vivo* (Dempsey et al., 2011). The decrease in SA observed in this study differed from the results of Ghosh et al. (2016) who reported an increase in SA at 6, 24, and 48 h after *A. thaliana* plants experienced a single, airborne tone for 1 h. This suggests that the response of *Arabidopsis* to complex, substrate borne vibrations can be quite different from responses to single, airborne tones. The decrease we measured in concentrations of most of the hormones in response to caterpillar feeding vibrations differs from those reported for real and simulated herbivory. For example, in a study of the local and systemic responses of *Nicotiana attenuata* to mechanical wounding and wounding plus oral secretions of *Manduca sexta*, levels of CKs, ABA, SA, OPDA, JA, and JA-Ile were unchanged or increased in response to both treatments, although carbon assimilation and stomatal conductance were reduced (Meza-Canales et al., 2017). This suggests that when provided separately, there is little overlap in phytohormone responses to feeding vibrations and phytohormone responses to mechanical damage + oral secretions. We know that pre-treatment of plants with feeding vibrations leads to priming of chemical defenses such that a later challenge with herbivory increases the levels of defensive chemicals above levels seen in unvibrated plants (Appel and Cocroft, 2014). How the signals arising from feeding vibrations, tissue removal, and oral secretions are integrated during normal herbivory remains an important unanswered question. Furthermore, since vibrational signals have faster transmission speeds through plants than electrical and phloem-borne signals, the integration of these signals over short periods of time to provide rapid systemic responses to stress is also unknown (Kollist et al., 2018). There is evidence from studies of *A. thaliana* roots that single tones can cause a rapid increase in cytosolic Ca^2+^, ROS, and K^+^ efflux (Rodrigo-Moreno et al., 2017). Examination of these phenomena in responses to insect feeding vibrations is an obvious next step.

One would not expect plant responses to insect feeding vibrations to exactly resemble those to actual insect feeding as the vibrations are only one of the cues involved in this interaction. Indeed, in actual insect feeding, the plant receives not only vibrations but also tissue damage and oral secretions of the insect. Plant responses to tissue damage from herbivory are distinct from those to mechanical wounding, in part because of the timing and extent of damage and the absence of insect oral secretions (Bricchi et al., 2010; Appel et al., 2014). Chewing insects commonly suppress the hydroperoxide lyase (HPL) branch of the oxylipin pathway and stimulate the allene oxide synthase (AOS) branch leading to JA synthesis (Savchenko et al., 2013). The JAZ repressors modulate many other plant hormone signaling pathways, effecting a wide range of physiological processes when de-repressed by JA-Ile (Howe et al., 2018).

### Activation of Classical Indirect Defenses Against Herbivores

Plant VOCs are used by a wide range of phytophagous, carnivorous, and parasitic insects to locate their plant or insect hosts (Arimura et al., 2009). This is the first study to examine the effect of feeding vibrations on VOC release, although there is a report of changes in VOCs in response to the related stimulus of touch (Markovic et al., 2018). The volatile profiles we measured for *A. thaliana* are different than those reported by others for two reasons. First, *A. thaliana* emits low levels of volatiles that are detectable only by sampling many plants. Since we were only able to vibrate one leaf on each of three plants in a volatile collection chamber, we were frequently near the limit of detection of many emitted compounds. Second, we limited our analysis to only those compounds we could quantify using commercially available standards because a preliminary comparison (Supplement 1) of the peak area (semi-quantitative) and standard curve (quantitative) methods of quantifying volatiles emitted by two plant species demonstrated significant differences in the two methods for several compounds.

At least five Lepidoptera, one Hymenoptera and two Coleoptera species have been shown to respond to MeSA, and seven Lepidoptera, one Hymenoptera, nine Hemiptera, one Diptera and three Coleoptera species responded to benzaldehyde (Bruce et al., 2005 and references herein). In the light of these results, the decrease in MeSA and benzaldehyde concentrations in response to insect feeding vibrations alone could be a way for the plant to decrease its attractiveness to herbivorous insects.

The concentration of β-ionone increased in response to vibration. β-ionone has been reported to deter feeding by the crucifer flea beetle (*Phyllotreta cruciferae*) and two species of mites (*Halotydeus destructo* and *Tetranychus urticae*), and to deter oviposition by silverleaf whiteflies (*Bemisia tabaci*) (Wang et al., 1999; Gruber et al., 2009; Wei et al., 2011; Caceres et al., 2016). The increase of β-ionone we reported in response to insect feeding vibrations could therefore reflect the activation of classical defenses against herbivores.

#### Alteration of Plant Metabolic Pathways

All phytohormone concentrations, except JA and IBA, were lower in plants that received the insect feeding vibrations, compared with plant that were subjected to the silent sham treatment. These changes could be explained by reduced synthesis, activation of alternative pathways, increased conjugation, increased catabolism, and/or translocation. We gathered no direct evidence concerning changes in metabolic or catabolic pathways, conjugation, or translocation. Our results do allow us to speculate about some factors that could explain the concentration changes we observed.

Vibration caused an increase in the JA concentration while reducing concentrations of OPDA, a precursor and JA-Ile, a conjugate. This pattern suggests that the JA increase did not come about *via* additional synthesis (Pratiwi et al., 2017), but could have arisen *via* the conversion of JA-Ile to JA (Kitaoka et al., 2011).

Phenylpropanoid pathway products benzaldehyde, SA, and MeSA were present at lower concentrations in vibrated plants than in control plants. Appel and Cocroft (2014) reported increases in the phenylpropanoid anthocyanins in response to vibration treatments. Metabolic tradeoffs between phenylpropanoid pools are frequently reported (Babst et al., 2014). Our current results suggest a possible tradeoff in substrate use between benzoic acid synthesis and anthocyanin production at the cinnamic acid step (Babst et al., 2014). In the auxin/glucosinolate biosynthesis pathway, concentrations of IBA increased while IAA concentration decreased in response to vibrations, suggesting an increase in the conversion of IAA into IBA (Strader et al., 2010).

Three of the four CKs measured, all four GAs, and ABA had lower concentrations in vibrated plants. All three classes share dimethylallyl pyrophosphate and/or isopentenyl pyrophosphate as precursors (Kanehisa Laboratories, 2016). We have no evidence to bear on the how this might have occurred, although the coincident lower concentrations suggest a downregulation of the pathway. Metabolites in the tetraterpenoid pathway (β-ionone and ABA) were also affected by the vibration treatment. The increase of β-ionone emission, while ABA concentration decreased, suggests a potential trade-off between those two routes in tetraterpenoid pathway (Cazzonelli and Pogson, 2010).

#### Similarities With Abiotic Response to Cold

The similarity of phytohormone responses to insect feeding vibrations and to cold suggests a functional similarity in how plants respond to these stresses. Why would insect feeding vibrations have an effect on plants similar to that of cold? The answer may reside in cold-induced changes in plant cell membranes and/or cytoskeleton. These cold-induced changes are thought to be transduced by the cytoskeleton, membrane-bound mechanoreceptors and/or focal adhesion complexes (Blume et al., 2017; Markovskaya and Shibaeva, 2017). Treatment of plants with individual phytohormones has been shown to cause changes in the stability and orientation of microtubules and actin filaments; however, studies that measure the effect of changes in phytohormone concentration on these structural elements during plant stress are lacking (reviewed in Blume et al., 2017). There is some evidence that mechanoreceptors are involved in transmission of cold signals across the plasma membrane. The Ca^2+^-permeable mechanosensitive channels MCA1 and MCA2 mediate cold-induced cytosolic Ca^2+^ increases and cold tolerance in *Arabidopsis* (Mori et al., 2018). Whether these mechanoreceptors are also sensitive to feeding vibrations is unknown.

In *A. thaliana*, the calcium fluxes and protein kinase cascades elicited by cold are thought to activate signaling pathways with regulatory networks that are highly co-regulated through extensive crosstalk (reviewed in Liu et al., 2018, 2019). There are also similarities in the expression of transcription factors known to respond to cold and to caterpillar feeding. Cold response genes (COR) are thought to be activated through three possible pathways. The best known involves the AP2/ERF transcription factors CBF1, CBF2, and CBF3 (also known as DREB1b, DREB1c, and DREB1a, respectively) which regulate cold tolerance and growth at low temperatures (Jia et al., 2016; Zhao et al., 2016). AP2/ERF transcription factors are known to be involved in hormone signaling and hormone-mediated stress responses (see review Xie et al., 2019). As mentioned above, the overexpression of these C-repeat binding factors caused a decrease in AUX levels and lower expression of genes associated with JA and SA signaling, and an increase in expression of genes associated with GA deactivation (Li et al., 2017), all changes consistent with our results.

The second pathway is CBF-independent and involves the higher expression of transcription factors whose expression is also induced by caterpillar feeding, such as ZAT10, ZAT12, MYB15, PIF3, and CCA1 (Rehrig et al., 2014; Zhao et al., 2016). Based on the likely need for organelle membrane remodeling in addition to that in the plasma membrane, there may also be a retrograde signaling pathway involving chloroplasts, mitochondria, and/or vacuoles, although this is largely unexplored.

Future experiments in our lab will explore the hormone profiles and co-expression of genes of plants responding to insect feeding vibrations and cold stress.

## Conclusion

Insect feeding vibrations cause changes in the volatiles released by leaves. They also cause lower concentrations of many phytohormone, including gibberellins, cytokinins, auxin, abscisic acid, salicylic acid, and several jasmonates, that resemble changes observed in plant responses to cold. Cold-associated changes in focal adhesion complexes, cell membranes and/or cytoskeleton are transduced by mechanosensors which are also a likely candidate for perception of insect feeding vibrations and the focus of our current research.

## Data Availability

All datasets generated for this study are included in the manuscript and/or the Supplementary Files.

## Author Contributions

MB, WN, RC, and HA designed the phytohormone and VOC experiments. CV, C-HL, JS, and HA designed and developed the VOC method. C-HL and DV helped with VOC analysis. WN performed the VOC experiments under the supervision of MB. MB helped with chemical analysis of the phytohormones. MB and RC did the statistical analyses. All the authors contributed to the manuscript.

## Conflict of Interest Statement

The authors declare that the research was conducted in the absence of any commercial or financial relationships that could be construed as a potential conflict of interest.
