# Supplementary material for: Caterpillar Chewing Vibrations Cause Changes in Plant Hormones and Volatile Emissions in Arabidopsis thaliana
Source: Front Plant Sci. 2019 Jun 26;10:810. doi: 10.3389/fpls.2019.00810 (PMC6607473; doi:10.3389/fpls.2019.00810)
Supplement: Supplementary file 1 [file Table_1.DOCX]

**Supplement 1. Comparison of a qualitative with a quantitative GC-MS method for plant volatile analysis.**

**Material and Methods**

***Plants, Insects, and Treatments***

*Arabidopsis thaliana* (Col-0 ecotype) (Brassicaceae) was used for this study because its genetic resources were needed as part of a larger study. Although the Col-0 ecotype has been reported to lack production of green leafy volatiles due to a 10-nucleotide deletion in the hydroperoxides lyase gene (At4g15440; Duan et al. 2005), subsequent studies have reported Col-0 to produce these compounds (Snoeren et al. 2009, 2010). A second plant species, *Brassica oleracea* (Flat Dutch variety, Walmart) (Brassicaceae) was used so that we could generalize our results to other species. Compared to *B. oleracea* plants which were sampled individually, *A. thaliana* is a weak emitter of VOCs so it was sampled in groups of three plants in one pot. Both species were grown from seed at 22 °C at 65 % relative humidity (RH) on an 8:16 light:dark photoperiod. At approximately four weeks post-germination, three *A. thaliana* plants were transplanted into larger pots to increase the leaf area for volatile sampling.

Third-instar *Pieris rapae* larvae (Lepidoptera, Pieridae) were obtained from our lab colony reared on *A. thaliana* on a 12:12 photoperiod under illumination of 180 μE⋅m^−2^⋅s^−1^ with 64 % RH.

Twenty replicates of similarly aged plants (seven weeks old) were used in our study, with each pot carefully wrapped with aluminum foil to minimize possible emissions from the pot and soil. Each run consisted of two replicates of the two following treatments: (1) control plants which received no damage and (2) herbivory plants treated with 20 *Pieris rapae* third-instar larvae. Volatiles were sampled for 24 hours for *A. thaliana* and 4 hours for *B. oleracea*.

***Volatile Collection System***

A dynamic, push-pull volatile collection system was use to trap emitted volatiles (Analytical Research Systems, Gainesville, FL). To remove any possible contaminants between trials, volatile chambers were cleaned with a solvent rinse of methanol and acetone, and volatile collection traps were conditioned at 300 °C for 15 minutes using helium as the carrier gas. Glass volatile collection traps contained 3 adsorbents to maximize the range of volatiles adsorbed: 20:35 Tenax-TA™, 60:80 Carboxen™ 1000, and 60:80 Carbosieve™ SIII (CDS Analytical Inc.). The volatile collection system delivered an outlet airflow rate between 17 and 18 PSI, a vacuum airflow rate between 18 and 20 PSI and an outlet airflow rate of 0.25 L/min.

***Selection of Volatiles to Quantitate, Ion Selection and Optimization***

To make the quantitative standard curve approach broadly useful, a thorough literature search was done to identify compounds commonly emitted by plants in response to both mechanical wounding and insect herbivory. From this list, 18 compounds with commercially available standards were chosen to include in the detection method. These compounds include: dimethyl sulfide, methyl jasmonate, jasmone, methyl salicylate, *cis*-3-hexenal, *cis*-3-hexen-1-ol, *cis*-3-hexenyl acetate, α-pinene, (S)-(-)-limonene, ocimene, β-caryophyllene, β-farnesene, 1-nonanol, 1-dodecanol, 1-tetradecanol, 3-pentanone, 1-penten-3-one, and 1-decanal. To build the method, we followed the general guidelines in Materić et al. (2015). For method building, 0.5, 1.0, 5.0, 10.0, 25.0, and 50.0 parts per million (ppm) solutions were made in 100 % acetonitrile for each of the compounds. Three microliters of each concentration were injected to construct the retention time (RT) profile and to identity quantitative ions for each compound (Figure 1; Table 1). The detection method was then made after constructing a calibration curve with each compound’s retention time and extracted ion spectrum. This method was then applied to each sample to determine the presence/absence of each compound, as well as the concentration of the compound in ppm. For each sample, all peaks identified by the software were also examined individually to confirm their identity based on retention time and quantification ions. Only those peaks with a signal to noise ratio greater than three (limit of detection, LOD; Table 1) and containing the appropriate fragment ions were considered to be confirmed peaks. Peak areas, and concentrations based on the quantitative standard curves, were then compared statistically (Table 2).

***GC-MS analysis of VOCs***

Plant volatiles were analyzed by thermal desorption gas chromatography-mass spectrometry (GC-MS). The samples were onto loaded onto a thermal desorption unit (TDU; ACEM 9300, CDS Analytical, Inc., Oxford, PA) in which the sample is volatilized and concentrated before being transferred to the GC. The identification and measurement of emitted volatiles was done by using a Varian 3400 GC coupled with a Varian Saturn 2200 ion-trap mass selective detector. The VOCs were desorbed by the TDU at 225 °C for 6 min to release volatiles from the collection tube and 250 °C for 7 min to concentrate them on the focusing trap. Samples were then injected into the Varian GC with a Hewlett Packard cross-linked methylsiloxane DB-5 capillary column (30 m, 0.25 mm, 1.00 µm) at 260 °C (split-injection mode with split ratio of 1:1000) with a 52.5 min oven temperature gradient of 35 °C for 10 min, 10 °C/min to 200 °C, 3 °C/min to 260 °C, and a hold for an additional 6 min. The trap, manifold, and transfer line temperatures were 220 °C, 50 °C, and 250 °C, respectively. The ionization energy (emission current) was 40 µamps, the maximum ionization time 25000 µsec, the ion scan range: 50-400 m/z, the ion storage level 45m/z and the pre-scan ionization time 100 µsec.

***Statistical analysis***

Statistical analyses were performed using R version 3.2.3 and RStudio version 0.99.467 (The R Foundation for Statistical Computing, Vienna, Austria). The comparison between control plants and plants with caterpillars, and the comparison of proportional changes between the two analytical methods (peak areas *versus* concentrations) were performed using Mann-Whitney tests. All proportional changes between control plants and plants infected by caterpillars are presented as average ± S.E.M.

**Results**

Using 18 commercially available standards for common plant VOCs (Figure 1), we developed a quantification method based on each compound’s retention time (RT) and quantification ions. The compounds exhibited a wide range of signal to noise ratios (S/N), limits of detection (LOD), and slopes (Table 1). We established standard curves for each compound and all slopes had R^2^ greater than 0.96 except for 1-tetradecanol which had an R^2^ of 0.86 (Table 1).

All 18 compounds were detected in the volatile emissions produced by one or both plant species under control conditions and in response to feeding by third-instar caterpillars of *P. rapae.* We calculated the amount of each compound based on peak area (semi-quantitative) and the standard curve developed with commercially available standards (quantitative) and these data are provided in Tables 1 and 2.

To determine whether the two methods produce similar results, for each, we calculated the percentage increase in emission of a compound in response to caterpillar feeding (Table 2). In *A. thaliana*, herbivory by *P. rapae* caused statistically significant increases in the amount of emitted *cis*-3-hexenal, *cis*-3-hexen-1-ol, and ocimene when calculated using the standard curve or peak area methods (Table 2). There was also a marginally significant increase in methyl salicylate (*p* < 0.1). No production of jasmone or 1-dodecanol by *A. thaliana* was detected, and the rest of the volatile compounds were emitted in measurable quantities but unaffected by caterpillar herbivory. All 18 volatile compounds were emitted by *B. oleracea*. Herbivory by *P. rapae* caused significant increases in the amount of emitted dimethyl sulfide, α-pinene, *cis*-3-hexenyl acetate, and methyl jasmonates when calculated using the standard curve or peak area methods (Table 2). There was also a marginally significant increase in *cis*-3-hexen-1-ol (*p* < 0.1).

Since the two methods gave qualitatively similar results, we then looked at how the two methods reported the magnitude of the proportional changes in response to herbivory (Figure 2; Table 3). In *A. thaliana*, the peak area method overestimated the amount of *cis*-3-hexenyl acetate, (S)-(-)-limonene, and methyl salicylate produced in response to herbivory by *P. rapae* compared with the standard curve method. In *B. oleracea*, the peak area method overestimated the amount of α-pinene and (S)-(-)-limonene, and underestimated the amount of dimethyl sulfide.

We then asked if discrepancies in amount reported by the two methods were related to the slopes of the standard curves, which may reflect differences in how the compounds react to the GC column matrix. Two of the compounds overestimated by the peak area method (α-pinene and (S)-(-)-limonene) have the highest slopes (Table 3). However, the slopes of *cis*-3-hexenyl acetate and methyl salicylate were much lower than those and well within the range of slopes of the other compounds (Table 3).

In conclusion, given the demonstrated overestimation of the amount of several volatiles by peak area method, we decided to proceed with the quantitative method and restrict our analysis of *A. thaliana* volatiles to only those for which we had commercially available standards.

**References**

Duan, H., Huang, M., Palacio, K., Schuler, M. 2005. Variations in CYP74B2 (hydroperoxide lyase) gene expression differentially affect hexenal signaling in the Columbia and Landsberg erecta ecotypes of Arabidopsis. *Plant Physiology* 139: 1529-1544.

Materić, D. B., Turner, C., Morgan, G., Mason, N., Gauci, V. 2015. Methods in plant foliar volatile organic compounds research. *Applications in Plant Sciences* 3: 1500044. doi:10.3732/apps.1500044

Snoeren, T. A., Van Poecke, R. M. P., Dicke, M. 2009. Multidisciplinary approach to unravelling the relative contribution of different oxylipins in indirect defense of *Arabidopsis thaliana*. *Journal of Chemical Ecology* 35: 1021-1031. doi: 10.1007/s10886-009-9696-3

Snoeren, T. A. L., Kappers, I. F., Broekgaarden, Co., Mumm, R., Dicke, M., Bouwmeester, H. J. 2010. Natural variation in herbivore-induced volatiles in *Arabidopsis thaliana*. *Journal of Experimental Botany* 61: 3041-3056. doi: 10.1093/jxb/erq127

**Table 1.** Characteristic features of the volatile organic compounds (VOCs) used in this study: retention time (RT), characteristic ions, signal/noise ratio (S/N), limit of detection (LOD; expressed in μg/m^3^), slope of the calibration curve and coefficient of determination of the regression curve of the standard curve (R^2^).

| **No.** | **VOCs** | **RT** | **Quantification ions** | **S/N** | **LOD** | **Slope** | **R^2^** |
| --- | --- | --- | --- | --- | --- | --- | --- |
| 1 | 1-penten-3-one | 7.99 | 54.8, 84.0 | 18 | 8.33 | 39,374 | 0.99 |
| 2 | 3-pentanone | 8.59 | 56.8, 86.0 | 11 | 13.64 | 81,261 | 0.99 |
| 3 | dimethyl sulfide | 11.70 | 60.8, 62.8, 62.1, 78.9, 81.0, 95.8 | 6 | 50.00 | 158,070 | 0.99 |
| 4 | *cis*-3-hexenal | 15.70 | 68.2, 82.8, 98.2, 68.8, 97.5 | 73 | 4.11 | 17,295 | 0.99 |
| 5 | *cis*-3-hexen-1-ol | 15.80 | 68.7, 81.8, 82.8, 69.5 | 260 | 1.15 | 2,863 | 0.98 |
| 6 | α-pinene | 18.00 | 92.8, 191.1, 193.1, 265.3, 267.2 | 928 | 0.32 | 576,820 | 0.96 |
| 7 | *cis*-3-hexenyl acetate | 19.45 | 81.8, 142.5, 141.5, 140.8 | 501 | 0.60 | 58,749 | 0.99 |
| 8 | (S)-(-)-limonene | 20.10 | 67.0, 81.0, 93.0, 95.0, 121.0, 136.9 | 5,224 | 0.06 | 752,330 | 0.96 |
| 9 | ocimene | 21.85 | 77.0, 79.0, 105.0, 121.0, 135.7, 136.9 | 4,236 | 0.07 | 67,648 | 0.99 |
| 10 | 1-nonanol | 22.45 | 56.0, 70.0, 83.0, 98.0 | 133 | 1.13 | 4,931 | 0.96 |
| 11 | 1-decanal | 22.99 | 57.0, 82.0, 95.0, 112.0 | 118 | 1.27 | 18,258 | 0.98 |
| 12 | methyl salicylate | 23.20 | 91.9, 121.0, 151.9 | 11,348 | 0.03 | 42,656 | 0.99 |
| 13 | jasmone | 26.40 | 166.0, 135.0, 131.0, 146.2, 91.0 | 11,437 | 0.03 | 10,971 | 0.99 |
| 14 | β-caryophyllene | 27.00 | 91.0, 108.9, 120.0, 132.9, 135.0, 147.0 | 2,876 | 0.10 | 64,813 | 0.99 |
| 15 | *trans*-β-farnesene | 27.07 | 68.8, 81.0, 93.0, 95.0, 133.0, 161.0 | 3,979 | 0.08 | 31,215 | 0.99 |
| 16 | 1-tetradecanol | 30.49 | 69.0, 83.0, 97.0, 111.0 | 16 | 9.38 | 12,555 | 0.86 |
| 17 | methyl jasmonate | 30.50 | 83.0, 93.0, 117.0, 135.0, 151.0, 167.0 | 217 | 1.38 | 6,359 | 0.99 |
| 18 | 1-dodecanol | 32.84 | 55.0, 69.0, 83.0, 97.0, 111.0, 140.0 | 13 | 11.54 | 65,880 | 0.99 |

**Table 2.** Increase in VOCs emitted by *A. thaliana* and *B. oleracea* in response to feeding by *P. rapae* caterpillars. The proportional increase in VOCs emitted by plants receiving herbivory compared to control plants was calculated using two methods, concentration (quantitative) *versus* peak area (semi-quantitative). Mann-Whitney statistical tests were conducted to compare control and infected plants. W = Wilcoxon rank statistic; ns (∙) = *p* ≤ 0.1; * = *p* ≤ 0.05; ** = *p* ≤ 0.01; *** = *p* ≤ 0.001.

| **VOCs** | ***Arabidopsis thaliana*** | | ***Brassica oleracea*** | |
| --- | --- | --- | --- | --- |
|  | **Concentration** | **Peak area** | **Concentration** | **Peak area** |
| 1-penten-3-one | W = 37.5, *p* = 0.809 | W = 37.5, *p* = 0.809 | W = 35.0, *p* = 0.772 | W = 35.0, *p* = 0.772 |
| 3-pentanone | W = 39.0, *p* = 0.911 | W = 39.0, *p* = 0.911 | W = 36.0, *p* = 0.590 | W = 39.5, *p* = 0.334 |
| dimethyl sulfide | W = 38.0, *p* = 0.823 | W = 38.0, *p* = 0.823 | W = 4.0, *p* = 0.004 ** | W = 4.0, *p* = 0.004 ** |
| *cis*-3-hexenal | W = 13.5, *p* = 0.005 ** | W = 13.5, *p* = 0.005 ** | W = 34.0, *p* = 0.862 | W = 34.0, *p* = 0.862 |
| *cis*-3-hexen-1-ol | W = 7.0, *p* = 0.002 ** | W = 7.0, *p* = 0.002 ** | W = 15.5, *p* = 0.090 (∙) | W = 15.5, *p* = 0.090 (∙) |
| α-pinene | W = 40.5, *p* = 1.000 | W = 41.5, *p* = 0.961 | W = 3.5, *p* = 0.003 ** | W = 3.0, *p* = 0.003 ** |
| *cis*-3-hexenyl acetate | W = 33.0, *p* = 0.546 | W = 33.0, *p* = 0.546 | W = 13.0, *p* = 0.050 * | W = 13.0, *p* = 0.050 * |
| (S)-(-)-limonene | W = 39.0, *p* = 0.929 | W = 38.0, *p* = 0.863 | W = 27.5, *p* = 0.674 | W = 27.0, *p* = 0.645 |
| ocimene | W = 9.0, *p* = 0.002 ** | W = 9.0, *p* = 0.002 ** | W = 27.0, *p* = 0.488 | W = 27.0, *p* = 0.488 |
| 1-nonanol | W = 45.0, *p* = 0.374 | W = 45.0, *p* = 0.374 | W = 28.0, *p* = 0.590 | W = 28.0, *p* = 0.590 |
| 1-decanal | W = 40.0, *p* = 1.000 | W = 40.0, *p* = 1.000 | W = 18.0, *p* = 0.161 | W = 18.0, *p* = 0.161 |
| methyl salicylate | W = 21.0, *p* = 0.092 (∙) | W = 21.0, *p* = 0.092 (∙) | W = 38.0, *p* = 0.563 | W = 38.0, *p* = 0.563 |
| jasmone | --- | --- | W = 28.0, *p* = 0.589 | W = 28.0, *p* = 0.590 |
| β-caryophyllene | W = 31.0, *p* = 0.424 | W = 31.0, *p* = 0.424 | W = 39.5, *p* = 0.432 | W = 39.5, *p* = 0.432 |
| *trans*-β-farnesene | W = 39.0, *p* = 0.911 | W = 30.5, *p* = 0.380 | W = 20.0, *p* = 0.142 | W = 20.0, *p* = 0.142 |
| 1-tetradecanol | W = 35.5, *p* = 0.678 | W = 33.0, *p* = 0.524 | W = 26.0, *p* = 0.574 | W = 26.0, *p* = 0.574 |
| methyl jasmonate | W = 33.5, *p* = 0.566 | W = 33.5, *p* = 0.566 | W = 53.0, *p* = 0.027 * | W = 53.0, *p* = 0.027 * |
| 1-dodecanol | --- | --- | W = 30.0, *p* = 0.848 | W = 30.0, *p* = 0.848 |

**Table 3.** Differences between the standard curve and peak area methods (proportional changes) in the magnitude of their reported increase in VOCs emitted by *A. thaliana* and *B. oleracea* in response to feeding by *P. rapae* caterpillars (see Figure 2). Mann-Whitney statistical tests were conducted to compare the two different analytical methods, concentration (quantitative) *versus* peak area (semi-quantitative). W = Wilcoxon rank statistic; ns (∙) = *p* ≤ 0.1; * = *p* ≤ 0.05; ** = *p* ≤ 0.01; *** = *p* ≤ 0.001.

| **VOCs** | ***Arabidopsis thaliana*** | ***Brassica oleracea*** |
| --- | --- | --- |
|  | **Proportional changes** | **Proportional changes** |
| 1-penten-3-one | W = 35.5, *p* = 0.663 | W = 29.0, *p* = 0.787 |
| 3-pentanone | W = 34.5, *p* = 0.594 | W = 26.0, *p* = 0.483 |
| dimethyl sulfide | W = 28.0, *p* = 0.268 | W = 9.0, *p* = 0.015 * |
| *cis*-3-hexenal | W = 35.5, *p* = 0.686 | W = 30.5, *p* = 0.914 |
| *cis*-3-hexen-1-ol | W = 55.5, *p* = 0.200 | W = 46.5, *p* = 0.141 |
| α-pinene | W = 22.5, *p* = 0.115 | W = 5.0, *p* = 0.003 ** |
| *cis*-3-hexenyl acetate | W = 16.0, *p* = 0.031 * | W = 16.0, *p* = 0.105 |
| (S)-(-)-limonene | W = 00.0, *p* = 0.000 *** | W = 7.0, *p* = 0.007 ** |
| ocimene | W = 23.0, *p* = 0.131 | W = 31.0, *p* = 0.945 |
| 1-nonanol | W = 41.0, *p* = 1.000 | W = 33.0, *p* = 0.945 |
| 1-decanal | W = 26.0, *p* = 0.222 | W = 34.0, *p* = 0.879 |
| methyl salicylate | W = 18.0, *p* = 0.050 * | W = 18.0, *p* = 0.161 |
| jasmone | --- | W = 33.0, *p* = 0.945 |
| β-caryophyllene | W = 21.0, *p* = 0.092 (∙) | W = 22.0, *p* = 0.315 |
| *trans*-β-farnesene | W = 18.0, *p* = 0.051 (∙) | W = 27.5, *p* = 0.666 |
| 1-tetradecanol | W = 35.0, *p* = 0.657 | W = 40.0, *p* = 0.442 |
| methyl jasmonate | W = 62.0, *p* = 0.063 (∙) | W = 49.0, *p* = 0.083 (∙) |
| 1-dodecanol | --- | W = 27.0, *p* = 0.613 |

**Figure 1.** Chromatogram of 18 VOC commercial standards from Table 1.


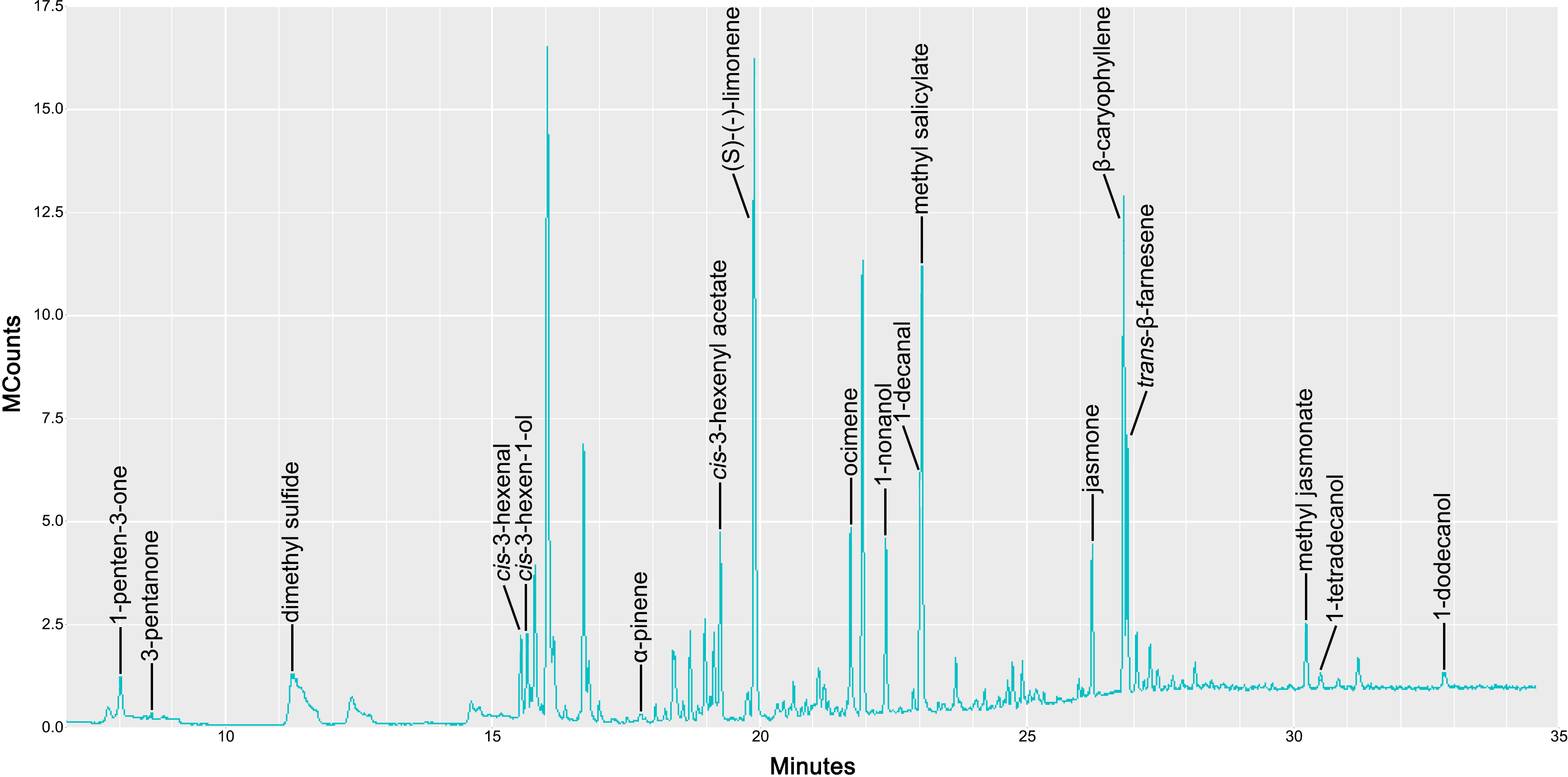
**Figure 2.** Differences between the standard curve and peak area methods in the magnitude of their reported increase in VOCs emitted by *A. thaliana* and *B. oleracea* in response to feeding by *P. rapae* caterpillars (average ± S.E.M.). Mann-Whitney statistical tests were conducted to compare two different analytical methods, concentration (quantitative) *versus* peak area (semi-quantitative). See Table 3 for statistical details: ns = no significant difference; ns (∙) = *p* ≤ 0.1; * = *p* ≤ 0.05; ** = *p* ≤ 0.01; *** = *p* ≤ 0.001.

**
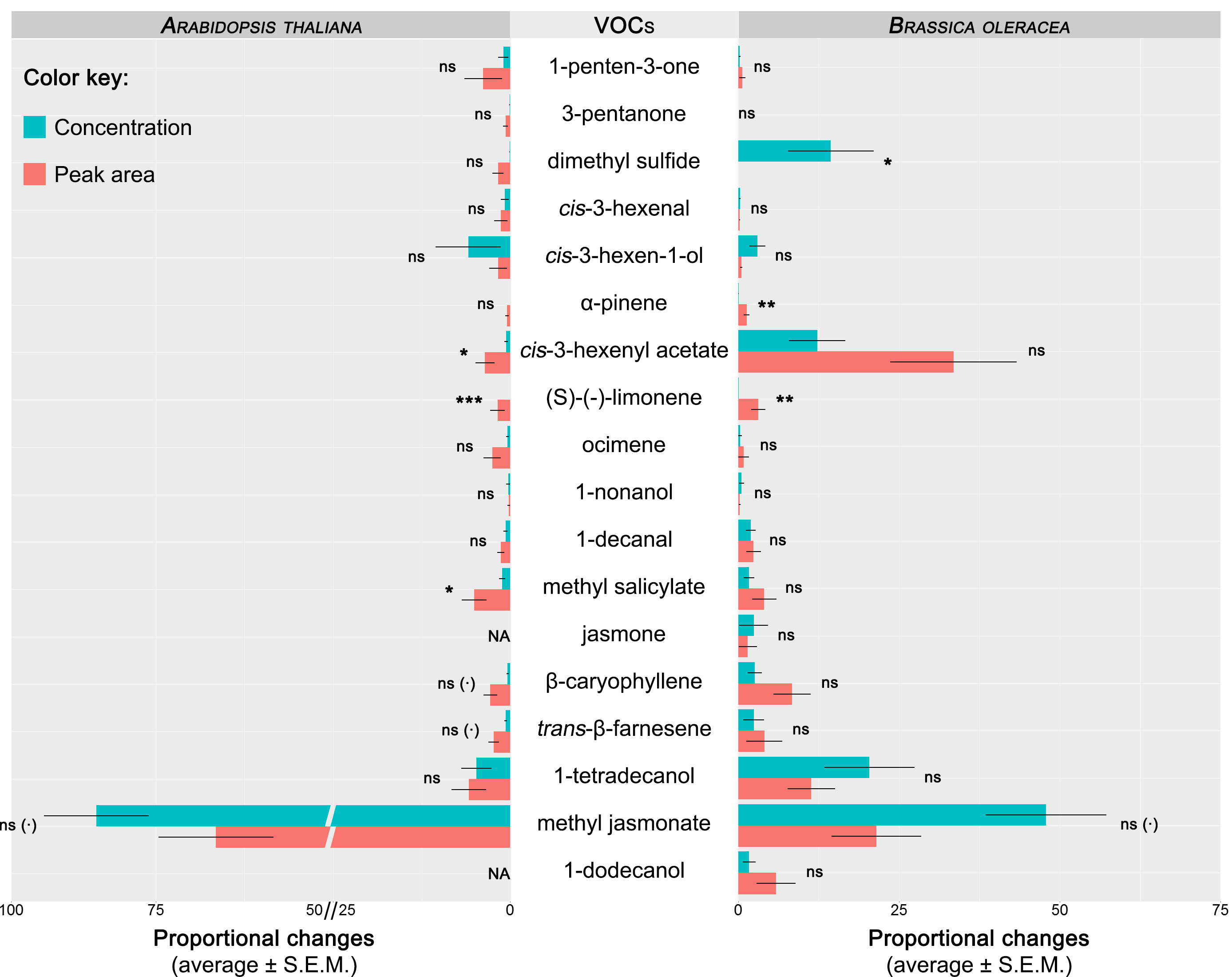
**
